# Black Soldier Fly Frass Combined with Ammonium Sulfate Enhances Maize Productivity and Nitrogen Use Efficiency Under Calcareous Soil Conditions

**DOI:** 10.3390/plants15182786

**Published:** 2026-09-11

**Authors:** Lasab Belachew Eshete, Augustine Talababie Phiri, Hongyan Zhang, Qihui Chen, Frank Mnthambala

**Affiliations:** 1 College of Resources and Environmental Sciences, China Agricultural University, Beijing 100193, China; lasabbelachew76@gmail.com (L.B.E.); augustinephiri8@gmail.com (A.T.P.); zhangzhy@cau.edu.cn (H.Z.); 2 Sanya Institute, China Agricultural University, Sanya 572025, China; 3 Beijing Food Safety Policy & Strategy Research Base, China Agricultural University, Beijing 100083, China; 4 Agrisciences Department, Mzuzu University, Mzuzu 105203, Malawi; mnthambala.f@mzuni.ac.mw; 5 SoilPlus Lab, Mzuzu University, Mzuzu 105203, Malawi

**Keywords:** black soldier fly frass, integrated nutrient management, nitrogen use efficiency, calcareous soils, maize productivity, soil fertility

## Abstract

Efficient nitrogen (N) management in calcareous soils remains challenging due to limited nutrient availability, poor synchronization between N supply and crop demand, and low fertilizer use efficiency. While black soldier fly frass (BSFF) represents an emerging organic amendment, its comparative effects relative to vermicompost (VC) and mineral N fertilizers under calcareous soil conditions remain insufficiently understood. In this study, we evaluated the effects of BSFF- and VC-based integrated organic–mineral fertilization strategies involving urea (U) and ammonium sulfate (AS) on maize productivity, nitrogen use efficiency (NUE), and selected soil chemical properties. A two-year field experiment was conducted at Quzhou Experimental Station, China Agricultural University, using a randomized complete block design (RCBD) with nine treatments: unfertilized control (CK), sole mineral fertilizers (U and AS), sole organic amendments (BSFF and VC), and integrated combinations (UBSFF, UVC, ASBSFF, and ASVC). All fertilizer treatments received 180 kg N ha^−1^ and were replicated three times. Our results showed that ASBSFF increased maize grain yield by 101.9–114.6% compared with CK across both growing seasons, while ASVC increased yield by 92.5–103.9%. ASBSFF also resulted in the highest N uptake, increasing it by 121.3% compared with CK, and achieved the highest agronomic efficiency (26.01–27.51 kg grain kg^−1^ N applied), apparent recovery efficiency (63.2%), and physiological efficiency (57.13–59.18 kg grain kg^−1^ N uptake). BSFF-based integrated treatments increased soil NH_4_^+^-N, NO_3_^−^-N, available phosphorus, and available potassium compared with VC-based treatments, likely due to differences in nutrient composition between BSFF and VC. Overall, combining BSFF with mineral N fertilizers, particularly AS, improved maize productivity and NUE in calcareous soils, demonstrating its potential as a sustainable nutrient management strategy.

## 1. Introduction

Crop production is essential for sustaining global food security, with nitrogen (N) being one of the most critical nutrients controlling crop growth and productivity [1]. However, improving N management remains a major challenge in modern agriculture because it directly influences crop yield, fertilizer use efficiency, and environmental sustainability [2]. Although N plays an essential role in plant physiological processes, including photosynthesis, protein synthesis, and biomass accumulation, a considerable proportion of applied N may be lost through processes such as ammonia volatilization, leaching, and denitrification [3]. These losses reduce nitrogen use efficiency (NUE) and contribute to environmental concerns, including greenhouse gas emissions and groundwater contamination [4]. Therefore, developing strategies that improve NUE while maintaining or increasing crop productivity is a key objective for sustainable agricultural systems.

The challenge of efficient N management is particularly pronounced in calcareous soils, which are widely distributed in arid and semi-arid regions [5]. These soils are commonly characterized by a high calcium carbonate content, alkaline pH, low organic matter, and limited nutrient availability [6,7]. Under such conditions, N availability can be affected by rapid transformation and loss processes, particularly ammonia volatilization under alkaline conditions, resulting in a reduced recovery of applied N by crops [8]. Furthermore, the high pH of calcareous soils can restrict the availability of other essential nutrients, particularly phosphorus (P) and micronutrients, thereby creating additional constraints for crop production [5,9]. As a result, farmers often depend heavily on synthetic N fertilizers to maintain yields, despite the fact that fertilizer efficiency remains relatively low under these soil conditions [10].

Conventional N fertilizers, including urea (U) and ammonium sulfate (AS), differ considerably in their transformation behavior after application, particularly in alkaline calcareous soils. Following hydrolysis, U can temporarily increase pH around fertilizer application sites, increasing the potential risk of ammonia volatilization under high-pH conditions [11]. In contrast, AS supplies ammonium-N together with sulfate, and its transformation through nitrification may influence N availability dynamics in soil. In addition, proton release during ammonium nitrification may locally modify soil chemical conditions in calcareous soils and potentially influence nutrient availability affected by alkaline conditions [12]. However, a sole reliance on mineral fertilizers does not address other soil fertility constraints, including low organic matter inputs and limited nutrient supply capacity, which are important for long-term soil fertility management [13].

In recent years, organic amendments have received increasing attention as complementary nutrient sources for improving soil fertility and enhancing fertilizer use efficiency [14]. Vermicompost (VC), produced through the decomposition of organic materials by earthworms, contains stabilized organic matter and plant-available nutrients [15]. Although previous studies have reported that VC application can improve soil nutrient availability, organic matter status, and nutrient release characteristics [16], nutrient release from VC is often relatively gradual, which may limit its ability to meet immediate crop nutrient requirements in intensive production systems.

Black soldier fly frass (BSFF), a by-product of insect-based organic waste recycling, has recently emerged as a potential organic fertilizer resource [17]. BSFF is nutrient-rich, containing N, phosphorus (P), and potassium (K), and possesses unique biochemical properties that may enhance its agronomic effectiveness [18]. While previous studies have suggested that BSFF-derived compounds, including chitin-related components, may influence microbial activity and nutrient transformation processes, these mechanisms were not measured in the present study and therefore remain hypothetical [19,20]. Compared with some conventional organic amendments, including VC, BSFF generally has a lower C/N ratio and higher concentrations of readily available nutrients, which may influence nutrient availability during crop growth [21]. Nevertheless, most previous studies have focused on BSFF application as an organic amendment or nutrient source under controlled conditions, while its performance when integrated with different mineral N sources under field conditions, particularly in calcareous soils, remains insufficiently understood.

Integrated nutrient management, which combines organic and mineral nutrient sources, has been widely proposed as a strategy to improve nutrient availability and sustain crop productivity [22]. By integrating the rapid nutrient supply from mineral fertilizers with additional nutrients and organic matter provided by organic amendments, such approaches may improve fertilizer performance under challenging soil conditions [23]. However, despite increasing interest in insect-derived organic fertilizers, limited field-based evidence is available regarding the effectiveness of BSFF when combined with different mineral N sources and compared with established organic amendments such as VC, particularly under alkaline calcareous soil conditions. Therefore, in this study, we evaluated the agronomic performance of BSFF integrated with mineral N fertilizers over two consecutive growing seasons under calcareous soil conditions. Specifically, we assessed maize growth, grain yield, N uptake, NUE indices, and selected post-harvest soil chemical properties under different combinations of organic amendments (BSFF and VC) and mineral N sources (urea and ammonium sulfate). We hypothesized that integrating BSFF or VC with mineral N fertilizers would improve maize productivity and NUE through complementary nutrient inputs and improved soil nutrient availability. However, because organic amendments differ in their nutrient composition, including P, K, and carbon inputs, the observed responses were considered to reflect the combined effects of amendment characteristics and fertilizer interactions rather than N supply alone. Although organic amendments may influence soil biological processes and N transformation pathways, these mechanisms were beyond the scope of the present field experiment.

## 2. Materials and Methods

### 2.1. Site Description and Soil Characterization

Our study was conducted at the Quzhou Experimental Station of China Agricultural University in Hebei Province, China (36°52′ N, 115°01′ E). The region has a warm–temperate monsoonal climate, with a mean annual precipitation of 542.8 mm and an average annual temperature of 13 °C. The soil at the experimental site is classified as calcareous fluvo-aquic soil with moderate salinity characteristics [24]. The surface layer (0–20 cm) consists of light loam, while the subsurface layer (20–40 cm) is sandy loam [25]. Climatic data for the experimental period of 2024 and 2025, including monthly mean temperature, total precipitation, and solar radiation, were obtained from the meteorological station located at the Quzhou Experimental Station. These data were used to characterize environmental conditions during the maize growing seasons. Prior to the initiation of the experiment in 2024, composite soil samples were collected from the 0–30 cm depth and analyzed for key physicochemical properties, including soil pH, electrical conductivity (EC), total nitrogen (N), available phosphorus (P), available potassium (K), and soil organic carbon (SOC) (Table 1).

### 2.2. Source of Fertilizers

Four fertilizer types were used in this study, comprising two organic amendments and two inorganic fertilizers: BSFF, VC, ammonium sulfate (AS), and urea (U). The organic amendments (BSFF and VC) were obtained from Wuhan Huier Biotechnology Co., Ltd., Wuhan, China, whereas AS and U were purchased from certified agro-dealers. Prior to application, BSFF and VC were analyzed for their chemical composition on a dry-weight basis. The analyses included total N, total P, and total K contents (Table 2). Total N was determined using the dry combustion method, while total P and total K were measured using the Egnér–Riehm method [26].

### 2.3. Experimental Design and Treatment Description

**Figure 1:**
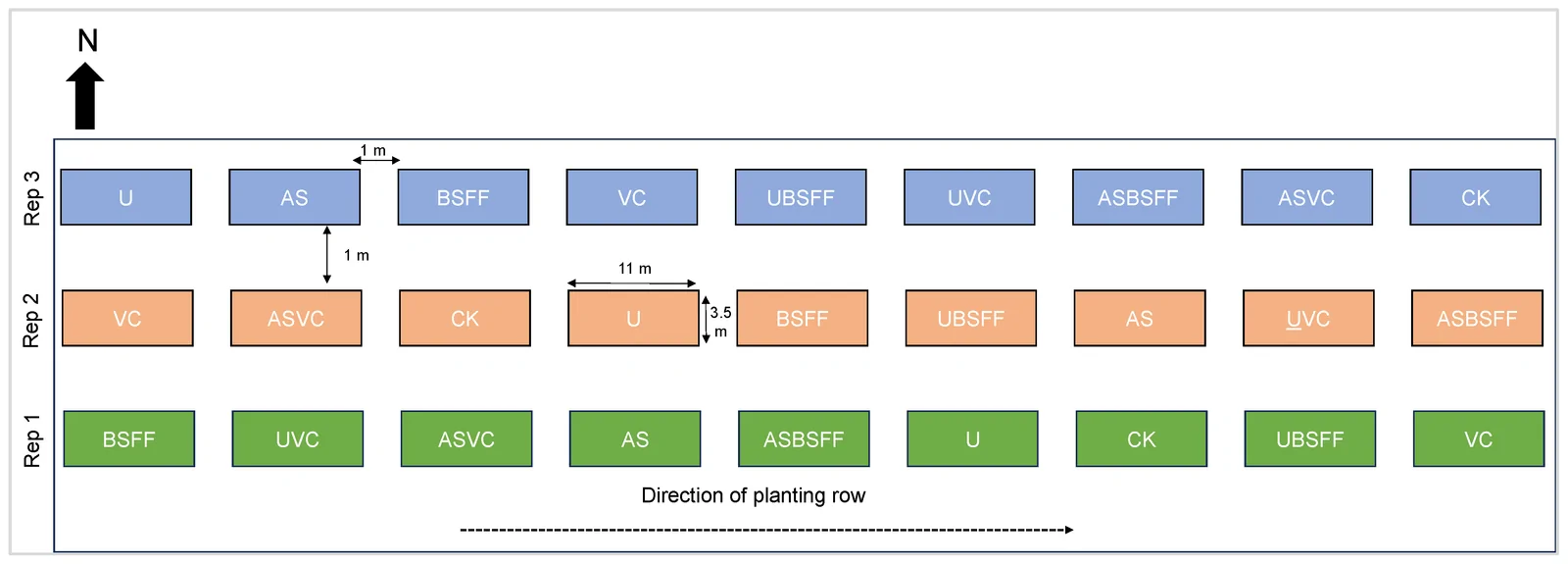
Experimental field design and plot layout. U, urea; AS, ammonium sulfate; BSFF, black soldier fly frass; VC, vermicompost; UBSFF, 50% urea + 50% BSFF at equal nitrogen proportions; UVC, 50% urea + 50% VC at equal nitrogen proportions; ASBSFF, 50% ammonium sulfate + 50% BSFF at equal nitrogen proportions; ASVC, 50% ammonium sulfate + 50% VC at equal nitrogen proportions; CK, no fertilization.

The field experiment was established on 4 June 2024, using a randomized complete block design (RCBD) with nine treatments: urea (U), ammonium sulfate (AS), black soldier fly frass (BSFF), vermicompost (VC), 50% urea + 50% BSFF (UBSFF), 50% urea + 50% VC (UVC), 50% ammonium sulfate + 50% BSFF (ASBSFF), 50% ammonium sulfate + 50% VC (ASVC), and an unfertilized control (CK). Each treatment was replicated three times (Figure 1). All nitrogen sources were applied at an equivalent rate of 180 kg N ha^−1^, which was selected based on recommended N management practices for summer maize production in the North China Plain. Organic amendments were applied according to their total N concentration to ensure that all fertilizer treatments received comparable N inputs. However, because organic amendments differ inherently in their nutrient composition, equivalent-N application resulted in different inputs of other nutrients, particularly P, K, and carbon (Table 3). Therefore, treatment responses were interpreted as the combined effects of N source, amendment characteristics, and associated nutrient inputs rather than N supply alone. The combined treatments were formulated on a N-equivalent basis, with each source contributing 50% of the total N. Organic amendments were incorporated into the top 15 cm soil layer one week before planting using a rotary tiller, whereas U and AS were applied in two splits: 80% at planting and 20% three weeks after planting. The experiment was conducted over two consecutive rainfed maize growing seasons, 4 June to 30 September 2024, and 20 June to 6 October 2025, hereafter referred to as the first and second years, respectively. The same plots were maintained throughout both growing seasons without treatment relocation. Each plot measured 11 m × 3.2 m. The spacing between plots was 0.5 m, and the distance between blocks was 1 m. The medium-maturing maize variety WC812 was used. Maize was planted at a spacing of 60 cm between rows and 25 cm between planting stations. All agronomic practices were carried out following standard smallholder farming practices.

### 2.4. Plant Data Collection

The growth and yield parameters of maize were recorded over two consecutive years. The variables measured included growth traits—plant height, mean single leaf area (MSLA), leaf area index (LAI), stem diameter, and leaf chlorophyll concentration—as well as yield components such as the number of grains per cob, 1000-grain weight, total dry biomass, and grain yield. Growth parameters were assessed at the tasseling stage. Plant height was measured from ground level to the tip of the uppermost fully extended leaf using a measuring tape. Stem diameter was determined using a digital caliper. For leaf area determination, three representative middle leaves per plant were selected. The length (from ligule to apex) and maximum width of each leaf were measured, and individual leaf area was calculated as leaf area = length × width × 0.75 [27]. Mean single leaf area (MSLA) was obtained by averaging the measured leaf areas. The leaf area index (LAI) was calculated as the ratio of total leaf area per plant to the ground area occupied by the plant. Leaf chlorophyll content was measured using a SPAD-502 chlorophyll meter, with three readings taken along each selected leaf (basal, middle, and distal sections), and the average value was recorded.

At physiological maturity, identified by the formation of a black layer at the base of maize kernels, plants were harvested from the net plot area (9 m × 2 m). The original plot size was 11 m × 3.5 m, and border effects were minimized by excluding 1 m from both ends of the plot length and 0.5 m from each side of the plot width. Grain yield and yield components were determined after harvest, and representative plant samples were collected for dry matter and N analysis. The number of grains per cob was determined by manually counting kernels from five representative cobs collected from each plot. The 1000-grain weight was determined by counting and weighing 1000 kernels using a precision balance. Grain yield was measured by harvesting all ears within the designated net plot area and was adjusted to a standard moisture content of 13%. Total plant biomass was estimated from five representative plants per plot, including aboveground and belowground components. Roots were carefully excavated and washed with tap water to remove adhering soil particles. Plant components were separated, air-dried, and subsequently oven-dried at 70 °C for 72 h until constant weight was achieved. The dry weights of all components were summed to determine total dry biomass per plant. Total biomass per hectare (kg ha^−1^) was calculated by multiplying dry biomass per plant by the corresponding plant population density and converting the value from grams to kilograms.

### 2.5. Plant Nutrient Analysis

Following harvest, plant components (leaves, stems, and grains) were separated, oven-dried at 70 °C for 72 h until a constant weight was achieved, and subsequently used for N concentration determination. The dried samples were ground into a fine powder using a laboratory mill and passed through a 1 mm sieve. Total N concentration was determined using the Kjeldahl method with concentrated sulfuric acid (H_2_SO_4_). N uptake per plant (g N plant^−1^) was calculated as the product of plant dry matter yield and nitrogen concentration (Equation (1)):

(1) $$N\;uptake\;({g\;N\;plant}^{-1})=\frac{Biomass\;\left(g\right)\times N\;concentration\;(\%)}{100},$$

Nitrogen use efficiency (NUE) was evaluated using specific indices, including agronomic efficiency (AE), apparent recovery efficiency (ARE), and physiological efficiency (PE). AE was calculated as follows:

(2) $$AE\;(kg\;grain\;{kg}^{-1}\;N\;applied=\frac{{(Grain\;yield}_{F}(kg\;{ha}^{-1})-{Grain\;yield}_{C}(kg\;{ha}^{-1})}{Quantity\;of\;N\;applied\;(kg\;N\;{ha}^{-1})},$$

where F represents fertilizer treatment, and C represents the control.

ARE was calculated as follows:

(3) $$ARE\;\left(\%\right)=\left(\frac{{N\;uptake}_{F}(kg\;N\;{ha}^{-1})-{N\;uptake}_{C}(kg\;N\;{ha}^{-1})}{Quantity\;of\;N\;applied\;(kg\;N\;{ha}^{-1})}\right)\times 100,$$

where F represents fertilizer treatment, and C represents the control.

PE was calculated as follows:

(4) $$PE\left({kg\;grain\;kg}^{-1}N\;uptake\right)=\frac{\left({Grain\;yield}_{F}\;(kg\;{ha}^{-1})-{Grain\;yield}_{C}(kg\;{ha}^{-1}\right)}{{N\;uptake}_{F}(kg\;N\;{ha}^{-1})-{N\;uptake}_{C}\;(kg\;N\;{ha}^{-1})},$$

where F represents fertilizer treatment, and C represents the control.

### 2.6. Soil Sampling and Analysis

Soil samples were collected after harvest from each treatment plot at a depth of 0–30 cm using a soil auger, following a simple random sampling method. Within each plot, soil was sampled from three points at the same depth (0–30 cm) and combined to form a composite sample. The composite samples were air-dried for the analysis of available P (mg kg^−1^), available K (mg kg^−1^), soil pH, and EC. Fresh (non-air-dried) soil samples were used for the determination of nitrate nitrogen (NO_3_^−^-N) and ammonium nitrogen (NH_4_^+^-N). All analyses were conducted at the Quzhou Experimental Station of China Agricultural University, China. Available P (Olsen-P) was determined using the Olsen extraction method. Briefly, soil samples were extracted with 0.5 M sodium bicarbonate (NaHCO_3_) solution (pH 8.5) at a soil-to-extractant ratio of 1:20 and shaken for 30 min. The extracts were filtered, and available P concentration was determined using the molybdenum blue colorimetric method. Available K was extracted and quantified using flame photometry [28]. Soil pH and EC were measured in a 1:2 (soil/water) suspension using calibrated pH and conductivity meters [28]. NO_3_^−^-N was determined through cadmium reduction followed by colorimetric analysis using a UV–visible spectrophotometer at 540 nm, and NH_4_^+^-N was measured using the indophenol blue method, with absorbance read at 655 nm [29].

### 2.7. Statistical Data Analysis

All statistical analyses were performed using R software (version 4.4.3, San Francisco, CA, USA: https://www.r-project.org/). Prior to analysis, data were checked for normality and homogeneity of variance using residual plots, the Shapiro–Wilk test, and Levene’s test. Where necessary, data were log-transformed to meet model assumptions, and back-transformed means are presented for clarity. A linear mixed-effects model was used to evaluate the effects of treatment, year, and their interaction on measured variables. Treatment and year were considered fixed effects, while block or replication was included as a random effect to account for the RCBD and repeated measurements on the same experimental units across years. When the interaction between treatment and year was not significant, main effects were interpreted. Mean separation was performed using estimated marginal means with Tukey’s adjustment at the 5% significance level. Pearson correlation analysis was conducted to examine relationships among soil chemical properties, nitrogen forms, and maize productivity variables. Principal component analysis (PCA) was performed to explore multivariate relationships among treatments and measured variables. Prior to PCA, all variables were standardized (centered and scaled) to ensure comparability. Principal components with eigenvalues greater than 1 were retained following the Kaiser criterion. The contribution of each variable to principal components was interpreted using loading values. All graphical outputs were generated using appropriate R packages.

## 3. Results

### 3.1. Climatic Conditions During the Experimental Period

**Figure 2:**
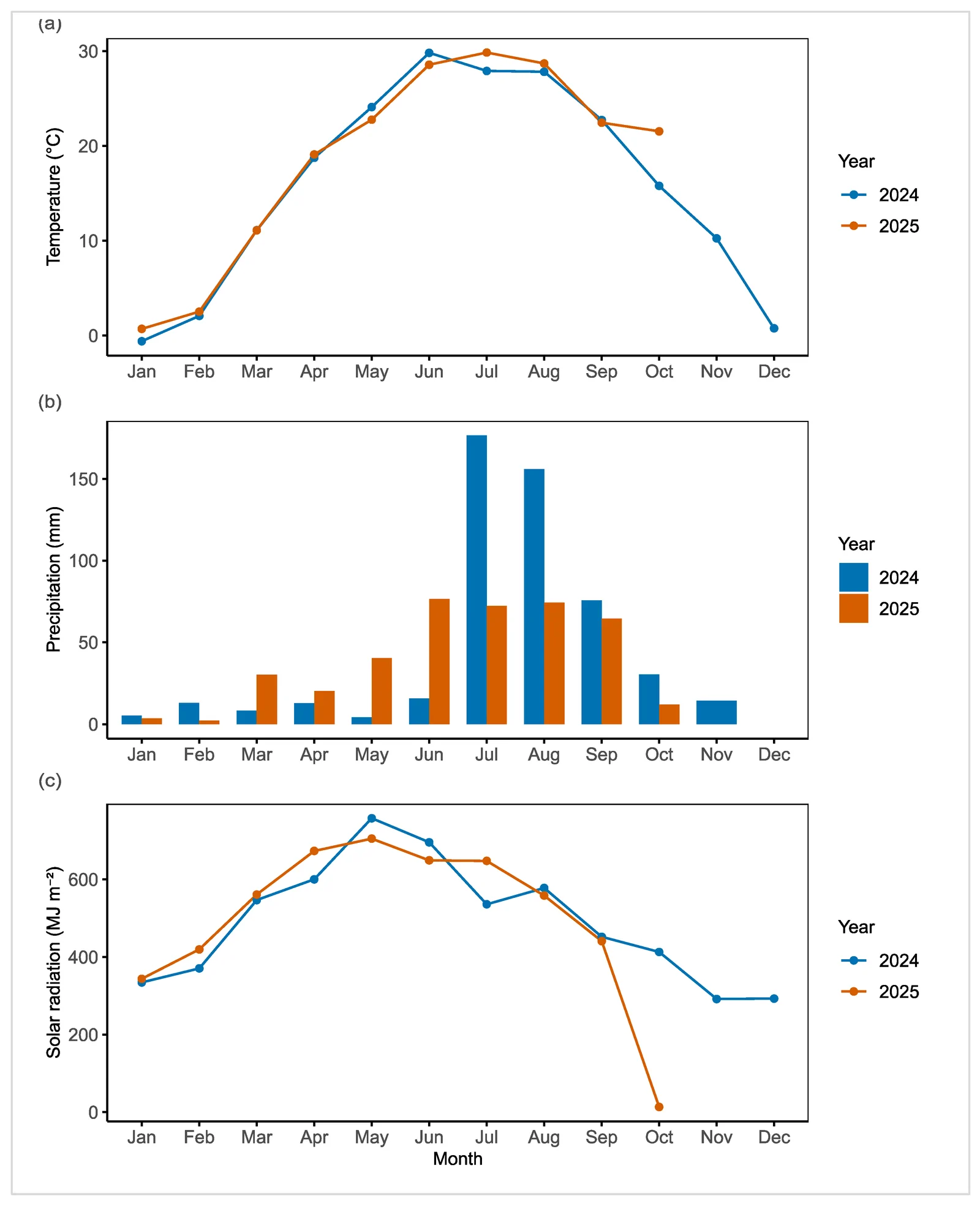
Monthly variation in climatic conditions at the experimental site in 2024 and 2025: (**a**) mean temperature, (**b**) total precipitation, (**c**) solar radiation.

Climatic conditions varied between the two growing seasons (Figure 2). Mean monthly temperature followed a typical seasonal pattern, increasing from early spring and peaking in June–July before declining toward the end of the year (Figure 2a). Temperatures were generally comparable between 2024 and 2025, although slightly higher values were observed during the mid-season in 2025. Precipitation showed a marked variability between years (Figure 2b). In 2024, rainfall was concentrated in July and August, with peak values exceeding those recorded in 2025. In contrast, 2025 exhibited a more evenly distributed rainfall pattern, with relatively higher precipitation occurring earlier in the growing season (May–June). Solar radiation also displayed seasonal trends, with maximum values occurring between May and July (Figure 2c). While radiation patterns were generally similar between years, 2025 showed slightly higher radiation during the early growth stages but a sharp decline later in the season compared to 2024. Overall, these variations in climatic conditions likely influenced maize growth dynamics and nutrient use efficiency across treatments.

### 3.2. Effects of Different Fertilizer Treatments on Maize Plant Growth Parameters

**Figure 3:**
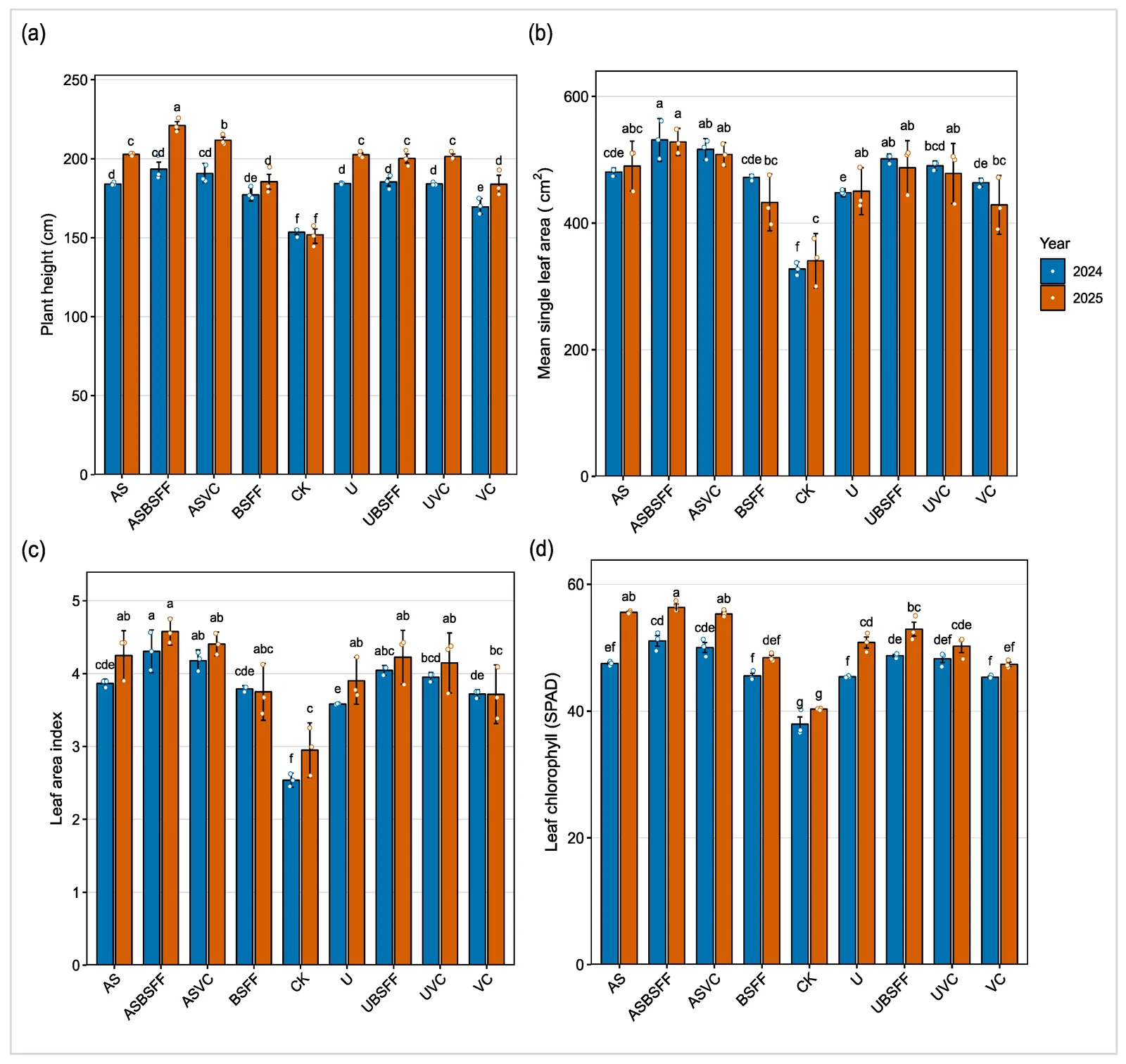
Maize plant height (**a**), mean single leaf area (**b**), leaf area index (**c**), and leaf chlorophyll (**d**) under different fertilizer treatments. Bars show mean ± SE. Different letters indicate significant differences among treatments (Tukey’s HSD, *p* < 0.05). U, urea; AS, ammonium sulfate; BSFF, black soldier fly frass; VC, vermicompost; UBSFF, 50% urea + 50% BSFF at equal nitrogen proportions; UVC, 50% urea + 50% VC at equal nitrogen proportions; ASBSFF, 50% ammonium sulfate + 50% BSFF at equal nitrogen proportions; ASVC, 50% ammonium sulfate + 50% VC at equal nitrogen proportions; CK, no fertilization.

Our results indicated that fertilizer treatments significantly influenced all measured growth parameters (*p* < 0.001), whereas year effects were generally non-significant, except for plant height, which showed a significant treatment × year interaction (*p* < 0.001) (Figure 3). Plant height varied significantly among treatments and growing seasons, with fertilizer treatments consistently producing taller plants than CK (Figure 3a). In 2025, ASBSFF resulted in the greatest plant height (213.45 cm), representing a 48.7% increase compared with CK (143.57 cm). Compared with sole organic amendment applications, ASBSFF increased plant height by 17.4% relative to BSFF (181.80 cm) and by 18.3% relative to VC (180.40 cm). Among integrated organic–mineral fertilizer treatments, ASBSFF, ASVC, UBSFF, and UVC generally resulted in greater plant height than sole organic amendment applications. Although BSFF and VC applied alone showed lower plant height than integrated fertilizer treatments, both maintained higher values than CK in both growing seasons.

Mean single leaf area (MSLA) was significantly affected by fertilizer treatments (Figure 3b). All fertilizer treatments increased MSLA compared with CK across both growing seasons. In 2024, ASBSFF recorded the highest MSLA (531.6 cm^2^), representing an increase of 18.7% compared with CK (447.5 cm^2^). Similarly, in 2025, ASBSFF resulted in the greatest MSLA (528.9 cm^2^), which was 16.0% higher than CK (455.9 cm^2^). Integrated organic–mineral fertilizer treatments generally resulted in greater MSLA values than sole organic amendment applications. Among sole organic amendments, BSFF maintained slightly higher MSLA values than VC in both years, with increases of 1.8% in 2024 (472.40 vs. 464.25 cm^2^) and 0.6% in 2025 (431.67 vs. 428.96 cm^2^). However, both organic amendments showed lower MSLA values than all integrated fertilizer treatments.

Similarly, leaf area index (LAI) was significantly affected by fertilizer treatments compared with CK in both growing seasons (Figure 3c). Integrated organic–mineral fertilizer treatments generally resulted in higher LAI values than sole organic amendment applications. ASBSFF produced the highest LAI values in both years (4.26 in 2024 and 4.55 in 2025), representing increases of 71.8% and 56.4%, respectively, compared with CK (2.48 in 2024 and 2.91 in 2025). In 2024, ASVC also showed a high LAI value (4.15), representing a 67.3% increase compared with CK. In 2025, ASVC (4.39), UBSFF (4.20), and UVC (4.11) increased LAI by 50.9%, 44.3%, and 41.2%, respectively, compared with CK. Among sole organic amendments, BSFF (3.76 in 2024 and 3.74 in 2025) and VC (3.71 in 2024 and 3.68 in 2025) did not differ significantly from each other, although BSFF showed slightly higher LAI values than VC by 1.3% and 1.6% in 2024 and 2025, respectively. Both organic amendments significantly increased LAI compared with CK, which consistently recorded the lowest values across both growing seasons.

Leaf chlorophyll content (SPAD) was significantly influenced by fertilizer treatments across both growing seasons (Figure 3d). Fertilizer treatments increased SPAD values compared with CK, with the highest chlorophyll content generally observed under integrated organic–mineral fertilizer treatments. ASBSFF recorded the highest SPAD values in both years (51.2 in 2024 and 56.9 in 2025), representing increases of 34.4% and 40.5%, respectively, compared with CK (38.1 in 2024 and 40.5 in 2025). Although sole BSFF (45.8 and 48.8) and VC (45.5 and 47.1) applications improved SPAD values compared with CK, their values remained lower than those observed under integrated fertilizer treatments. Among sole organic amendments, BSFF showed slightly higher SPAD values than VC, with increases of 0.7% in 2024 and 3.6% in 2025, respectively.

### 3.3. Effects of Different Fertilizer Treatments on Maize Yield and Yield Components

**Figure 4:**
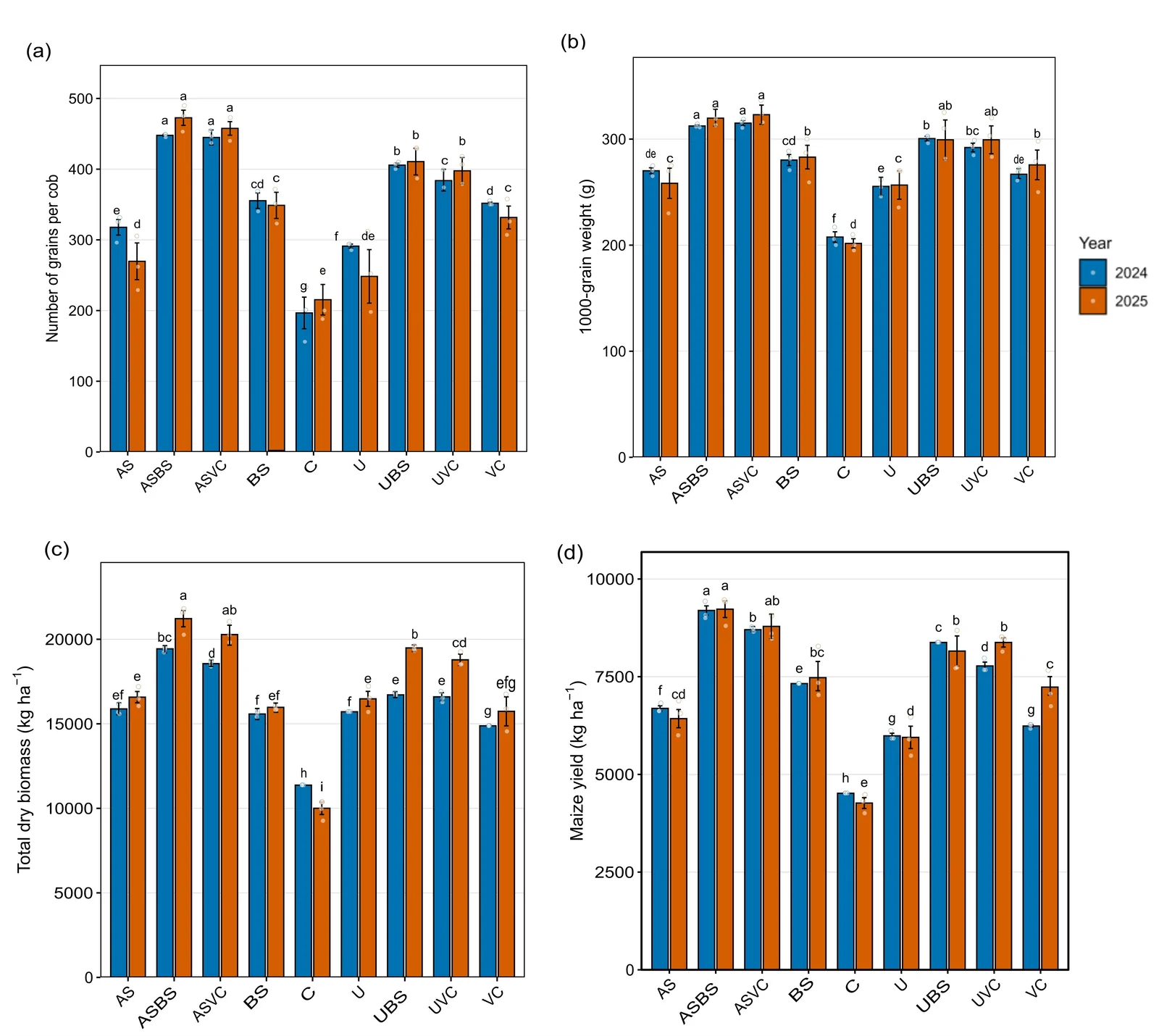
Number of grains per cob (**a**), 1000-grain weight (**b**), total dry biomass (**c**), and grain yield (**d**) under different fertilizer treatments. Bars show mean ± SE. Different letters indicate significant differences among treatments (Tukey’s HSD, *p* < 0.05). U, urea; AS, ammonium sulfate; BSFF, black soldier fly frass; VC, vermicompost; UBSFF, 50% urea + 50% BSFF at equal nitrogen proportions; UVC, 50% urea + 50% VC at equal nitrogen proportions; ASBSFF, 50% ammonium sulfate + 50% BSFF at equal nitrogen proportions; ASVC, 50% ammonium sulfate + 50% VC at equal nitrogen proportions; CK, no fertilization.

Maize yield and yield components were significantly influenced by fertilizer treatments (*p* < 0.001), whereas year effects were generally non-significant, with similar treatment trends observed across both growing seasons. Total biomass showed a significant treatment × year interaction (*p* = 0.013) (Figure 4).

The number of grains per cob increased significantly under fertilizer treatments compared with CK (Figure 4a). Integrated organic–mineral fertilizer treatments produced the highest grain numbers, with ASBSFF recording 442.83 and 465.45 grains per cob in 2024 and 2025, respectively. These values represented increases of 125.2% and 123.9%, respectively, compared with CK (196.63 and 207.90 grains per cob). This was followed by ASVC (440.31 and 451.57 grains per cob), which increased grain number by 123.9% and 117.2% in 2024 and 2025, respectively, compared with CK. Other integrated treatments, including UBSFF and UVC, also increased grain number relative to CK, although their values were lower than those observed under ASBSFF and ASVC. Among sole organic amendments, BSFF resulted in numerically higher grain numbers than VC in both years, while both organic amendments produced significantly greater grain numbers than CK.

A similar trend was observed for 1000-grain weight (Figure 4b). Fertilizer treatments significantly increased 1000-grain weight compared with CK across both growing seasons. The highest values were recorded under integrated organic–mineral fertilizer treatments, with ASBSFF producing 336.58 g in 2024 and 343.83 g in 2025, representing increases of 53.6% and 60.5%, respectively, compared with CK (219.12 g in 2024 and 214.20 g in 2025). ASVC showed comparable performance, with 1000-grain weights of 333.96 and 340.33 g in 2024 and 2025, respectively, which were slightly lower than ASBSFF by 0.8% and 1.0%. Other integrated treatments, including UBSFF and UVC, also resulted in higher 1000-grain weights than CK. Sole organic amendments produced intermediate values, with BSFF (297.28 and 300.68 g) showing slightly higher values than VC (283.19 and 293.27 g), by 5.0% and 2.5% in 2024 and 2025, respectively. Overall, both organic amendments increased 1000-grain weight compared with CK, although their effects were lower than those observed under integrated organic–mineral fertilizer treatments.

Total dry biomass was significantly influenced by the interaction between fertilizer treatments and year (Figure 4c). In 2025, ASBSFF produced the highest biomass (21,442.68 kg ha^−1^), representing an increase of 110.7% compared with CK (10,177.86 kg ha^−1^). ASVC (20,652.17 kg ha^−1^) and UBSFF (19,960.47 kg ha^−1^) showed comparable biomass production, although they were 3.7% and 6.9% lower than ASBSFF, respectively. Compared with CK, ASVC and UBSFF increased biomass by 102.9% and 96.1%, respectively. Other integrated treatments and sole organic amendments, including BSFF (16,205.53 kg ha^−1^) and VC (15,909.09 kg ha^−1^), resulted in intermediate biomass values, with BSFF showing a slightly higher biomass production than VC by 1.9%. All fertilizer treatments produced greater biomass than CK, and biomass values were generally higher in 2025 than in 2024 across fertilizer treatments.

Grain yield was significantly influenced by fertilizer treatments but not by year (Figure 4d). The highest grain yields were observed under ASBSFF (9075.63 kg ha^−1^ in 2024 and 9201.68 kg ha^−1^ in 2025), representing increases of 101.9% and 114.6%, respectively, compared with CK (4495.80 kg ha^−1^ in 2024 and 4285.71 kg ha^−1^ in 2025). ASVC also produced high yields (8655.46 and 8739.49 kg ha^−1^ in 2024 and 2025, respectively), representing increases of 92.5% and 103.9% compared with CK, while remaining slightly lower than ASBSFF by 4.6% and 5.0% in the two seasons, respectively. These treatments were followed by UBSFF and UVC, which showed intermediate yields between integrated fertilizer treatments and sole organic amendments. Sole organic amendments produced intermediate yields, with BSFF (7310.92 and 7478.99 kg ha^−1^) showing higher grain yields than VC (6344.54 and 7226.89 kg ha^−1^) by 15.2% and 3.5% in 2024 and 2025, respectively. Across all treatments, CK consistently recorded the lowest grain yield. Although the year effect was not statistically significant, grain yield values were generally higher in 2025 than in 2024 across most treatments.

### 3.4. Effects of Different Fertilizer Treatments on Total Nitrogen Uptake and Nitrogen Use Efficiency

**Figure 5:**
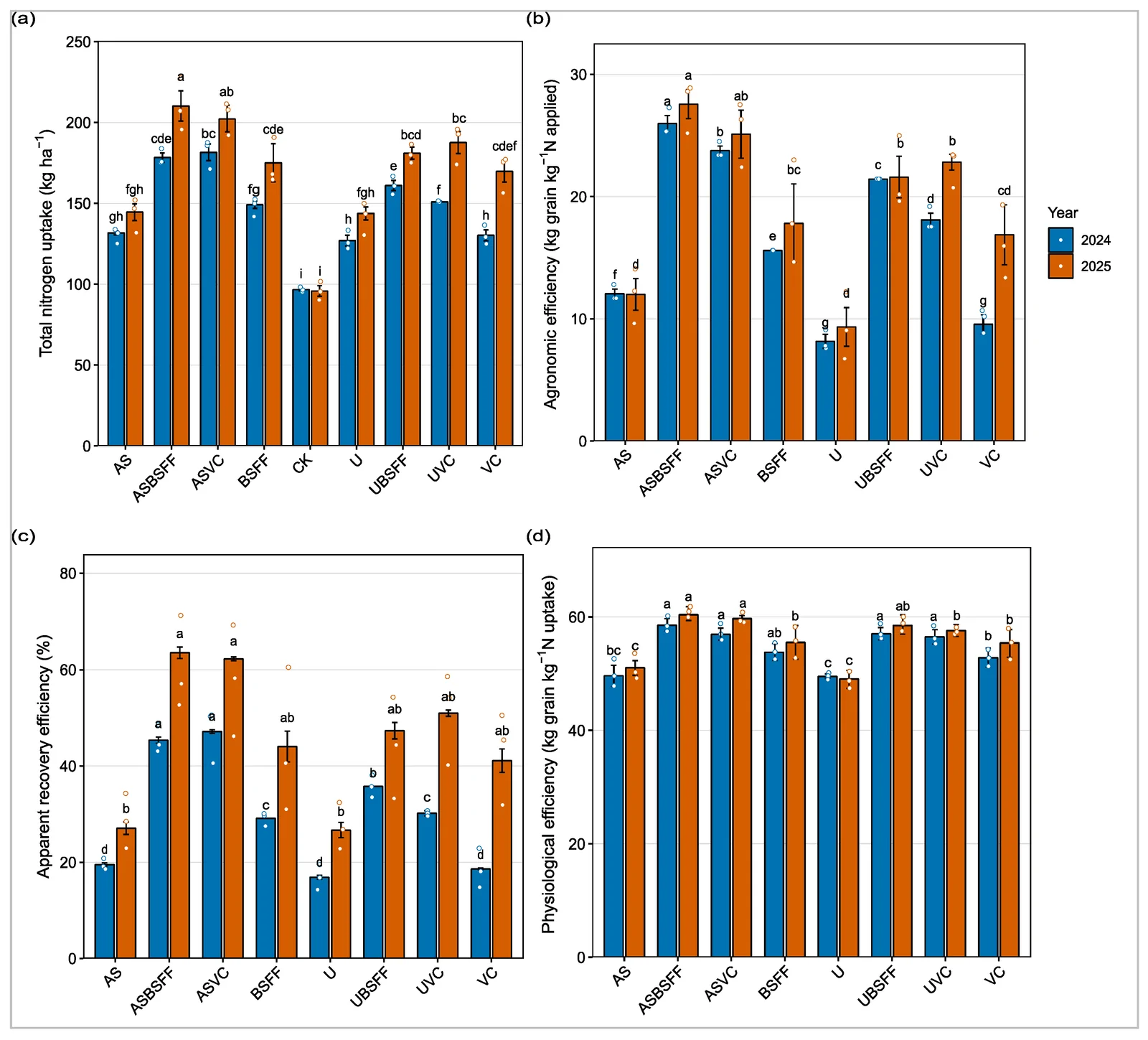
Maize total nitrogen uptake (**a**), agronomic efficiency (**b**), apparent recovery efficiency (**c**), and physiological efficiency (**d**) under different fertilizer treatments. Bars show mean ± SE. Different letters indicate significant differences among treatments (Tukey’s HSD, *p* < 0.05). U, urea; AS, ammonium sulfate; BSFF, black soldier fly frass; VC, vermicompost; UBSFF, 50% urea + 50% BSFF at equal nitrogen proportions; UVC, 50% urea + 50% VC at equal nitrogen proportions; ASBSFF, 50% ammonium sulfate + 50% BSFF at equal nitrogen proportions; ASVC, 50% ammonium sulfate + 50% VC at equal nitrogen proportions; CK, no fertilization.

Total N uptake was significantly influenced by the interaction between fertilizer treatments and year (*p* < 0.001) (Figure 5a). In 2025, ASBSFF recorded the highest N uptake (211.45 kg ha^−1^), representing an increase of 121.3% compared with CK (95.83 kg ha^−1^). ASVC (204.16 kg ha^−1^) also resulted in significantly higher N uptake, representing an increase of 113.0% compared with CK, while being slightly lower than ASBSFF by 3.4%. These treatments were followed by UBSFF and UVC, which resulted in higher N uptake than sole organic amendment applications. Sole organic amendments, including BSFF (176.04 kg ha^−1^) and VC (169.79 kg ha^−1^), showed lower N uptake values but remained significantly higher than CK, with BSFF showing a 3.7% higher N uptake than VC. Overall, N uptake was generally higher in 2025 than in 2024 for most treatments, whereas CK showed a slight decline.

Agronomic efficiency (AE) was significantly influenced by fertilizer treatments (*p* < 0.001), whereas no significant effect of year was observed (Figure 5b). ASBSFF recorded the highest AE values in both years (26.01 and 27.51 kg grain kg^−1^ N applied in 2024 and 2025, respectively), representing increases of 9.9% and 10.0% compared with ASVC (23.67 and 25.02 kg grain kg^−1^ N applied, respectively). Compared with sole organic amendment applications, ASBSFF increased AE by approximately 65.8% and 55.1% relative to BSFF, and by 174.7% and 61.9% relative to VC in 2024 and 2025, respectively. Other integrated treatments, including UBSFF (21.42 and 21.54 kg grain kg^−1^ N applied) and UVC (17.98 and 22.63 kg grain kg^−1^ N applied), also showed higher AE values than sole organic amendments, including BSFF (15.69 and 17.74 kg grain kg^−1^ N applied) and VC (9.47 and 16.99 kg grain kg^−1^ N applied). Overall, integrated organic–mineral fertilizer treatments exhibited greater AE values than sole organic amendment applications.

Similarly, ARE was significantly affected by fertilizer treatments (*p* = 0.001), whereas no significant effect of year was observed (Figure 5c). The integrated treatments ASBSFF (45.3% in 2024 and 63.2% in 2025) and ASVC (48.2% in 2024 and 61.8% in 2025) recorded the highest ARE values. In 2025, ASBSFF achieved a slightly higher ARE than ASVC by 1.4 percentage points. These treatments were followed by UVC (41.2% in 2024 and 50.5% in 2025) and UBSFF (35.9% in 2024 and 47.5% in 2025). Sole organic amendments, including BSFF (28.8% in 2024 and 42.4% in 2025) and VC (18.3% in 2024 and 39.5% in 2025), resulted in lower ARE values. However, BSFF maintained higher ARE values than VC by 10.5 and 2.9 percentage points in 2024 and 2025, respectively.

Physiological efficiency (PE) was significantly affected by fertilizer treatments (*p* = 0.009), whereas the effect of year was not significant (Figure 5d). Across both growing seasons, the highest PE values were observed under integrated organic–mineral fertilizer treatments, particularly ASBSFF (57.13 kg grain kg^−1^ N uptake in 2024 and 59.18 kg grain kg^−1^ N uptake in 2025) and ASVC (56.06 and 58.09 kg grain kg^−1^ N uptake in 2024 and 2025, respectively). ASBSFF achieved slightly higher PE values than ASVC by 1.9% in both years, corresponding to increases of 1.07 and 1.09 kg grain kg^−1^ N uptake in 2024 and 2025, respectively. These treatments were followed by UBSFF (55.69 and 57.73 kg grain kg^−1^ N uptake in 2024 and 2025, respectively) and UVC (55.47 and 56.64 kg grain kg^−1^ N uptake in 2024 and 2025, respectively), which also maintained relatively high PE values. Sole organic amendments, including BSFF (52.37 and 54.42 kg grain kg^−1^ N uptake) and VC (51.74 and 54.71 kg grain kg^−1^ N uptake), resulted in intermediate PE values and showed no significant difference from each other. Although year effects were not significant, PE values were generally slightly higher in 2025 than in 2024 for most treatments.

### 3.5. Effects of Different Fertilizer Treatments on Selected Soil Chemical Properties

**Figure 6:**
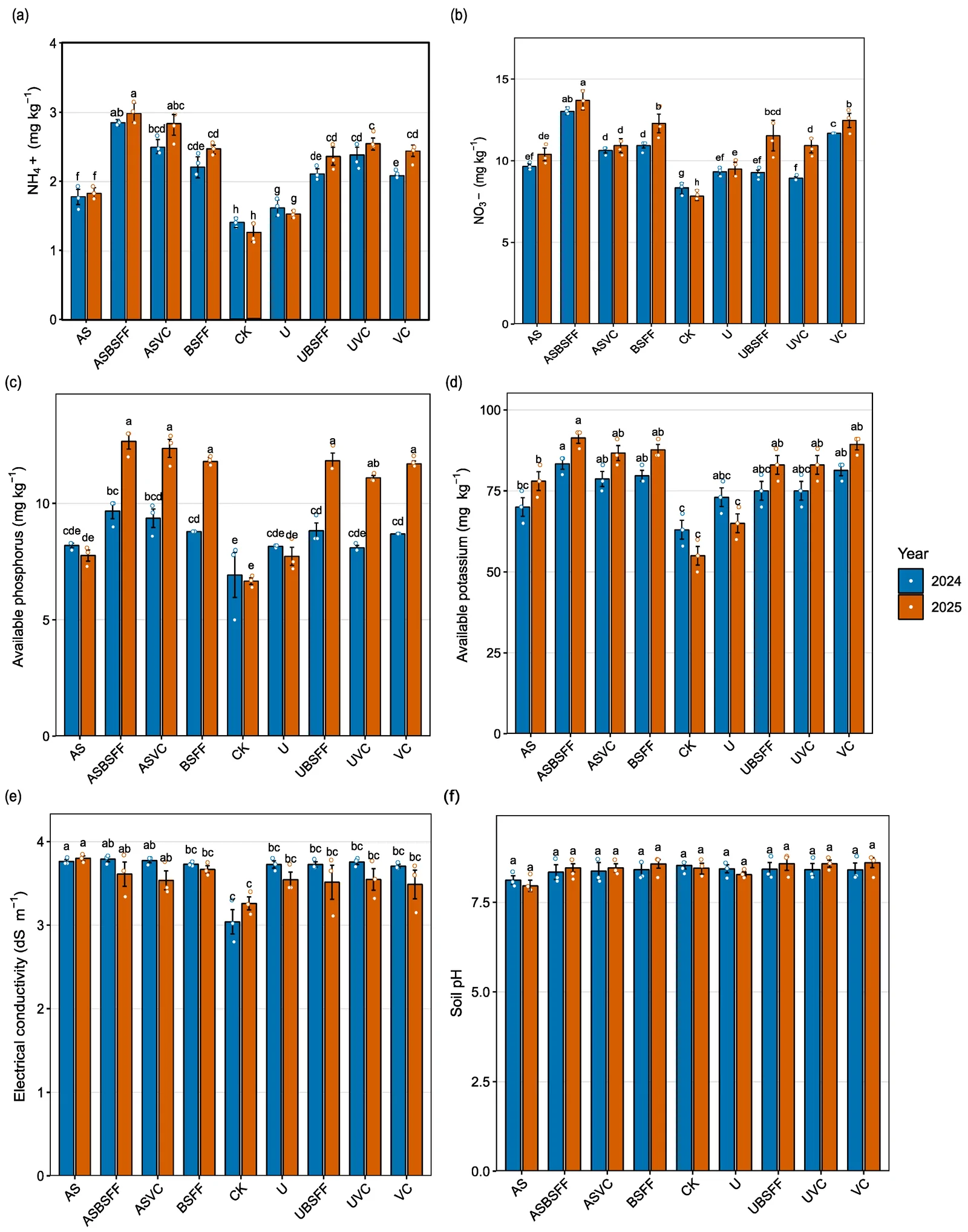
Ammonium (**a**), nitrate (**b**), available phosphorus (**c**), available potassium (**d**), electrical conductivity (**e**), and soil pH (**f**) under different fertilizer treatments. Bars show mean ± SE. Different letters indicate significant differences among treatments (Tukey’s HSD, *p* < 0.05). NH_4_^+^ = ammonium; NO_3_^−^ = nitrate. U, urea; AS, ammonium sulfate; BSFF, black soldier fly frass; VC, vermicompost; UBSFF, 50% urea + 50% BSFF at equal nitrogen proportions; UVC, 50% urea + 50% VC at equal nitrogen proportions; ASBSFF, 50% ammonium sulfate + 50% BSFF at equal nitrogen proportions; ASVC, 50% ammonium sulfate + 50% VC at equal nitrogen proportions; CK, no fertilization.

Soil NH_4_^+^–N, NO_3_^−^–N, and available P were significantly affected by the interaction between fertilizer treatments and year (*p* < 0.001), whereas available K was significantly influenced by both fertilizer treatments (*p* < 0.001) and year (*p* = 0.037), with no significant interaction effect. Soil EC was significantly affected by fertilizer treatments (*p* = 0.005), while soil pH was not significantly influenced by either fertilizer treatments (*p* = 0.103) or year (*p* = 0.176) (Figure 6). Soil NH_4_^+^–N concentrations were significantly affected by the interaction between fertilizer treatments and year (Figure 6a). In 2025, the highest NH_4_^+^–N concentration was observed under ASBSFF (3.01 mg kg^−1^), representing an increase of 137.0% compared with CK (1.27 mg kg^−1^). ASVC also resulted in a high NH_4_^+^–N concentration (2.84 mg kg^−1^), which was 123.6% higher than CK but 5.6% lower than ASBSFF. In 2024, ASBSFF recorded 2.52 mg kg^−1^, showing increased NH_4_^+^–N availability compared with CK. Across both years, integrated treatments (UBSFF and UVC), as well as sole organic amendments, resulted in higher NH_4_^+^–N concentrations than CK. Among sole organic amendments, BSFF (2.49 mg kg^−1^) showed a higher NH_4_^+^–N concentration than VC (2.14 mg kg^−1^) by 16.4%.

Similarly, soil NO_3_^−^–N concentrations were significantly affected by the interaction between fertilizer treatments and year (Figure 6b). The highest NO_3_^−^–N concentration was observed under ASBSFF in 2025, reaching 13.64 mg kg^−1^, which represented an increase of 77.4% compared with CK (7.69 mg kg^−1^). Sole VC application resulted in 12.52 mg kg^−1^ NO_3_^−^–N, which was 62.8% higher than CK but 10.0% lower than ASBSFF. Similarly, UBSFF (11.65 mg kg^−1^) and BSFF (12.27 mg kg^−1^) increased NO_3_^−^–N concentrations by 51.5% and 59.6%, respectively, compared with CK. ASVC and UVC showed relatively lower NO_3_^−^–N concentrations than ASBSFF, BSFF, UBSFF, and VC. The CK treatment consistently recorded the lowest NO_3_^−^–N concentrations across both growing seasons. Overall, NO_3_^−^–N concentrations tended to be higher in 2025 than in 2024 for most fertilizer treatments, whereas CK showed higher values in 2024 (8.55 mg kg^−1^) than in 2025 (7.68 mg kg^−1^).

Available P concentrations were significantly influenced by fertilizer treatments and year, with no significant treatment × year interaction effect (Figure 6c). The highest available P concentrations were recorded under integrated organic–mineral fertilizer treatments, particularly ASBSFF (12.97 mg kg^−1^) and ASVC (12.81 mg kg^−1^), representing increases of 88.8% and 86.5%, respectively, compared with CK (6.87 mg kg^−1^). Other integrated treatments, including UBSFF (9.11 mg kg^−1^) and UVC (11.52 mg kg^−1^), showed intermediate available P concentrations. Sole organic amendments also resulted in higher available P concentrations than CK, with BSFF (12.10 mg kg^−1^) showing a slightly higher value than VC (11.97 mg kg^−1^) by 1.1%. Available P concentrations were higher in 2025 compared to 2024.

Available K concentrations were significantly influenced by fertilizer treatments and year, with no significant treatment × year interaction effect (Figure 6d). Across both growing seasons, ASBSFF recorded the highest available K concentrations (83.23 mg kg^−1^ in 2024 and 91.30 mg kg^−1^ in 2025), representing increases of 32.7% and 65.2%, respectively, compared with CK (62.73 mg kg^−1^ in 2024 and 55.28 mg kg^−1^ in 2025). Similar high available K concentrations were observed under VC (81.37 mg kg^−1^ in 2024 and 89.44 mg kg^−1^ in 2025), BSFF (80.12 mg kg^−1^ in 2024 and 87.57 mg kg^−1^ in 2025), and ASVC (78.26 mg kg^−1^ in 2024 and 86.34 mg kg^−1^ in 2025). Other integrated treatments, including UBSFF (75.15 and 83.23 mg kg^−1^) and UVC (75.15 and 82.61 mg kg^−1^), also maintained higher available K concentrations than CK. Overall, available K concentrations were generally higher in 2025 than in 2024 across fertilizer treatments.

Soil EC was significantly influenced by fertilizer treatments, but not by year (Figure 6e). The highest EC values were observed under sole AS, which recorded 3.81 and 3.83 in 2024 and 2025, respectively, representing increases of 26.2% and 17.1% compared with CK. Similarly, ASBSFF (3.79 in 2024 and 3.60 in 2025) and ASVC (3.78 and 3.56 in 2024 and 2025, respectively) showed higher EC values than CK, with increases of 25.5% and 10.1%, and 25.2% and 8.9%, respectively. Other treatments, including BSFF, U, UBSFF, UVC, and VC, exhibited comparable EC values and showed no significant differences from ASBSFF, ASVC, or CK. The CK treatment generally recorded the lowest EC values. Although year effects were not statistically significant, EC values tended to be slightly higher in 2024 than in 2025 across most treatments, except for AS.

Soil pH was not significantly influenced by fertilizer treatments or year (Figure 6f). Although no significant differences were detected, numerical variations were observed among treatments. Sole organic amendments (BSFF and VC) tended to maintain relatively higher pH values than integrated organic–mineral fertilizer treatments, sole mineral fertilizer treatments, and CK, with BSFF showing slightly higher values than VC. In contrast, sole mineral fertilizer treatments (AS and U) generally showed comparatively lower pH values. Across years, soil pH showed only minor fluctuations. Under sole mineral fertilizer treatments and CK, pH values tended to be slightly higher in 2024 than in 2025, whereas organic amendments and integrated treatments generally showed slightly higher pH values in 2025 than in 2024.

### 3.6. Relationships Among Soil Chemical Properties and Maize Productivity Under Different Fertilizer Treatments

**Figure 7:**
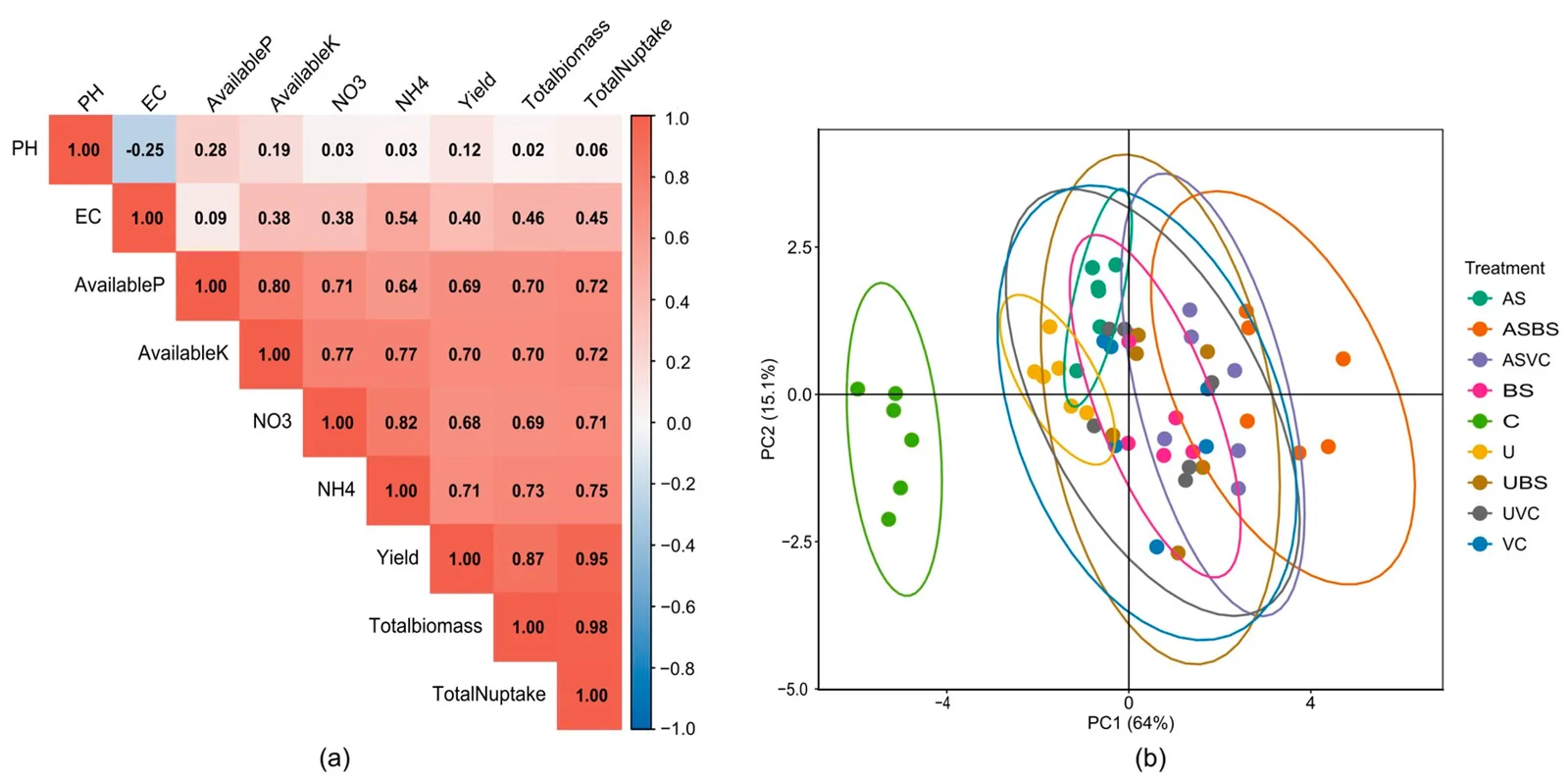
Relationships among soil chemical properties and maize productivity, and principal component analysis of treatments. (**a**) Pearson correlation matrix showing relationships among soil pH, electrical conductivity (EC), available phosphorus (P), available potassium (K), nitrate (NO_3_^−^), ammonium (NH_4_^+^), grain yield, total biomass, and total N uptake. Color intensity represents the strength and direction of correlations. (**b**) Principal component analysis showing the distribution of treatments based on soil chemical properties and plant variables. PC1 and PC2 explain 64% and 15% of the total variation, respectively. Ellipses indicate the dispersion of treatments. U, urea; AS, ammonium sulfate; BSFF, black soldier fly frass; VC, vermicompost; UBSFF, 50% urea + 50% BSFF at equal nitrogen proportions; UVC, 50% urea + 50% VC at equal nitrogen proportions; ASBSFF, 50% ammonium sulfate + 50% BSFF at equal nitrogen proportions; ASVC, 50% ammonium sulfate + 50% VC at equal nitrogen proportions; CK, no fertilization.

The correlation analysis revealed significant relationships among soil chemical properties, nitrogen forms, and maize productivity variables (Figure 7a). Grain yield showed strong positive correlations with total biomass (r = 0.87) and total N uptake (r = 0.95), while total biomass was also strongly correlated with total N uptake (r = 0.98), indicating that crop productivity was closely associated with plant biomass accumulation and N acquisition. Soil available nutrients showed strong positive associations, with available P positively correlated with available K (r = 0.80), NO_3_^−^-N (r = 0.71), and NH_4_^+^-N (r = 0.62). Similarly, available K showed positive correlations with NO_3_^−^-N and NH_4_^+^-N, while NO_3_^−^-N and NH_4_^+^-N were strongly correlated (r = 0.82). Soil EC showed moderate positive correlations with NH_4_^+^-N (r = 0.54), NO_3_^−^-N (r = 0.38), and available K (r = 0.38). In contrast, soil pH showed weak relationships with most variables, consistent with the absence of significant treatment and year effects on soil pH. Overall, the correlation patterns indicate that nutrient availability and plant N acquisition were closely associated with maize productivity, although these relationships do not establish causal mechanisms.

Principal component analysis (PCA) showed that PC1 and PC2 explained 64% and 15% of the total variation, respectively, accounting for 79% of the overall variability among fertilizer treatments (Figure 7b). The contribution and loading patterns of individual variables to PC1 and PC2 are presented in Table S1. Variables associated with maize productivity, including grain yield, total biomass, and total N uptake, together with soil nutrient indicators such as available P, available K, NH_4_^+^-N, and NO_3_^−^-N, showed strong contributions to PC1. Therefore, PC1 represented an integrated gradient associated with soil nutrient availability and maize productivity. Treatments located on the positive side of PC1, particularly ASBSFF and ASVC, were associated with higher nutrient availability and crop productivity, whereas CK was positioned on the negative side of PC1, reflecting lower soil nutrient status and maize performance. Other fertilizer treatments, including UBSFF, UVC, BSFF, VC, AS, and U, showed intermediate positions along PC1, indicating moderate effects on soil nutrient conditions and crop productivity. PC2 explained an additional 15% of the total variation and represented secondary differences among fertilizer treatments, mainly associated with variations in soil chemical characteristics, particularly EC and pH. The separation of treatments along PC2 suggested additional treatment-related variation beyond the major nutrient availability–productivity gradient represented by PC1. Overall, PCA demonstrated that integrated organic–mineral fertilizer treatments were more closely associated with improved soil nutrient availability and maize productivity, whereas the CK showed contrasting characteristics.

## 4. Discussion

### 4.1. Effects of Different Fertilizer Treatments on Maize Crop Performance

Integrated organic–mineral fertilization significantly improved maize growth, yield components, and grain yield compared with sole fertilizer applications. Among all treatments, ASBSFF consistently produced the highest maize productivity, demonstrating the potential agronomic advantage of combining a mineral N source with an organic nutrient source. The improved performance of integrated treatments may reflect complementary nutrient supply patterns, whereby mineral fertilizers provide readily available N while organic amendments contribute additional nutrients and carbon inputs [22]. However, because BSFF and VC were applied on an equivalent-N basis rather than an equivalent total nutrient basis, the treatments received different amounts of P, K, and carbon. BSFF supplied greater amounts of P, K, and carbon than VC at the same N application rate, which may have contributed to the higher productivity observed under BSFF-based treatments. Therefore, the superior performance of BSFF should be interpreted as a combined response to its nutrient composition, carbon inputs, nutrient release characteristics, and interaction with mineral fertilizers rather than as an effect of N supply alone.

The higher plant height, leaf area index, chlorophyll content, grain number per cob, and 1000-grain weight observed under integrated treatments indicate improved crop growth and reproductive development [30]. These responses are consistent with previous studies showing that integrated nutrient management can improve crop productivity by increasing nutrient availability and reducing the limitations associated with sole fertilizer application, particularly in nutrient-constrained soils [31]. The stronger performance of ASBSFF compared with ASVC indicates that the type and composition of the organic amendment can influence the agronomic performance of integrated fertilizer strategies. However, because BSFF and VC supplied unequal amounts of accompanying nutrients under the equivalent-N application strategy, the higher performance of BSFF-based treatments cannot be attributed exclusively to amendment type. BSFF generally had a lower C/N ratio and higher concentrations of P and K than VC, which may have contributed to greater nutrient availability during crop growth [32,33]. In contrast, the relatively higher C/N ratio and more stabilized organic matter in VC may be associated with slower nutrient release [21,34]. Thus, the observed advantage of BSFF represents an integrated response to differences in nutrient composition and amendment characteristics. The superior performance of ASBSFF may also reflect the contrasting characteristics of the mineral N sources under the alkaline conditions of the calcareous soil used in this study. Urea hydrolysis can temporarily increase pH around fertilizer application sites, potentially increasing the risk of ammonia volatilization under alkaline conditions [35,36]. Ammonium sulfate, in contrast, supplies ammonium-N together with sulfate, and subsequent nitrification can release protons that may locally reduce alkalinity [37]. These characteristics may contribute to differences in N availability between ammonium sulfate- and urea-based treatments. However, because N loss pathways and soil N transformation processes were not directly measured in the present study, these mechanisms remain plausible explanations rather than experimentally demonstrated processes.

The variation between growing seasons further demonstrates the influence of environmental conditions on fertilizer performance. Differences in rainfall distribution and temperature between 2024 and 2025 may have affected nutrient dissolution, soil nutrient availability, and crop nutrient uptake. In addition, because the same plots were maintained across seasons, residual effects from previous fertilizer applications may have contributed to differences between years [38]. These findings highlight that fertilizer performance under field conditions is determined by interactions among fertilizer characteristics, soil conditions, and seasonal climate variability.

### 4.2. Effects of Different Fertilizer Treatments on Total Nitrogen Uptake and Nitrogen Use Efficiency

Nitrogen uptake and NUE are important indicators for evaluating fertilizer management strategies [39]. In this study, integrated organic–mineral fertilization increased N uptake and NUE indices, including AE, ARE, and PE, compared with sole fertilizer applications. Among the integrated organic–mineral treatments, ASBSFF consistently showed the highest values, indicating greater agronomic recovery and utilization of applied N under combined organic and mineral nutrient management. The higher N uptake under integrated treatments suggests that combining mineral fertilizers with organic amendments improved the availability of nutrients during crop growth [40]. Mineral fertilizers provide readily available N that supports early crop development, whereas organic amendments may contribute additional nutrients through gradual nutrient release [41]. While this complementary nutrient supply pattern may have contributed to improved N availability and crop N acquisition [42], these processes were not directly measured in the present study. The higher NUE observed under BSFF-based treatments compared with VC-based treatments may be related to differences in amendment characteristics. BSFF contained a lower C/N ratio and higher nutrient concentrations, including P and K, than VC, which may have influenced nutrient availability during the growing season [18]. However, because BSFF and VC supplied different amounts of accompanying nutrients under the equivalent-N application strategy, the higher NUE observed in BSFF-based treatments should be interpreted as a combined response to amendment composition and nutrient inputs rather than an effect of N source alone.

The improvement in NUE under integrated treatments is particularly relevant for calcareous soils, where fertilizer N efficiency can be influenced by alkaline conditions and potential N loss pathways such as ammonia volatilization and nitrate movement. Previous studies have shown that ammonium sulfate and urea differ in their transformation dynamics in alkaline soils, which may influence N availability and fertilizer recovery [36,37]. The acidifying effect associated with ammonium nitrification may also locally modify soil chemical conditions in calcareous soils. However, because ammonia volatilization, nitrate leaching, N_2_O emissions, mineralization rates, and microbial processes were not directly measured in this study, these mechanisms remain possible explanations rather than experimentally demonstrated processes. Therefore, the higher NUE indices observed under ASBSFF may be associated with improved nutrient availability and interactions between mineral and organic nutrient sources, but further studies using direct measurements of N transformation and loss pathways are required to confirm these mechanisms.

Seasonal differences also influenced N dynamics: variations in rainfall distribution and temperature between the two growing seasons may have affected fertilizer dissolution, nutrient availability, and crop uptake [43]. The greater response observed under different climatic conditions highlights the importance of considering environmental variability when assessing NUE responses under field conditions.

### 4.3. Effects of Different Fertilizer Treatments on Selected Soil Chemical Properties

Integrated organic–mineral fertilization improved measured soil nutrient availability, particularly NH_4_^+^-N, NO_3_^−^-N, available P, and available K, compared with sole fertilizer applications. These changes indicate that combining organic amendments with mineral fertilizers can improve soil nutrient supply conditions during crop production [44]. The higher NH_4_^+^-N concentrations observed in BSFF-containing treatments indicate greater residual ammonium availability after harvest, although the processes responsible for this pattern were not directly measured [45]. In calcareous soils, ammonium is generally less mobile than nitrate, and the presence of residual NH_4_^+^-N may contribute to continued mineral nitrogen availability within the soil system [46]. Meanwhile, increased NO_3_^−^-N concentrations indicate ongoing nitrification under integrated treatments, suggesting the coexistence of ammonium and nitrate forms that provide different mineral N pools for plant uptake [47]. Integrated treatments also increased available P, which is particularly important in calcareous soils, where P availability is frequently limited by calcium-associated fixation processes [48].

The higher P availability observed under BSFF-based treatments may be partly explained by the greater P inputs supplied by BSFF compared with VC. In addition, organic amendments may influence P availability through interactions between organic matter inputs and soil P dynamics. However, processes such as organic compound release, phosphate competition for adsorption sites, and calcium-phosphate transformations were not measured in this study and require further investigation. The increase in available K under integrated treatments likely resulted from direct K inputs supplied by organic amendments and nutrient release during decomposition [49]. The higher nutrient concentration of BSFF compared with VC may partly explain the greater P and K availability observed in BSFF-based treatments. However, because organic amendments were applied on an equivalent-N basis, differences in accompanying nutrient inputs should be considered when interpreting these treatment effects.

Soil EC was highest under sole AS application, indicating greater soluble salt accumulation compared with integrated treatments. The lower EC values observed following organic amendment addition suggest that organic–mineral combinations influenced soluble salt dynamics under the studied conditions. However, longer-term studies are required to determine whether these changes persist across multiple cropping seasons. Soil pH was not significantly altered by fertilizer treatments, which is consistent with the strong buffering capacity of calcareous soils [50]. Although organic amendment treatments showed slightly higher pH values than sole mineral fertilizer treatments, these differences were insufficient to overcome the inherent buffering characteristics of the soil. Therefore, the observed productivity improvements were more likely associated with differences in nutrient availability rather than short-term changes in soil pH.

Correlation analysis and PCA further supported the relationship between soil nutrient status and crop performance. The positive associations among nutrient availability, N uptake, biomass, and grain yield suggest that improved nutrient conditions were important factors associated with enhanced maize productivity. In contrast, the weak relationship between soil pH and productivity variables indicates that short-term pH variation played a limited role in crop responses under this calcareous soil.

### 4.4. Limitations and Practical Implications

Although integrated BSFF–mineral fertilizer management improved maize productivity, N uptake, and measured soil nutrient availability, several limitations should be considered when interpreting these findings. First, organic amendments were compared on an equivalent-N basis, resulting in unequal P, K, and carbon inputs among treatments. Although this approach reflects practical fertilizer application scenarios, it limits our ability to attribute treatment differences solely to N source or amendment identity. Future studies should consider nutrient-balanced experimental designs, including P and K compensation treatments, to separate amendment-specific effects from differences in nutrient inputs. Second, this study did not measure soil microbial biomass, enzyme activities, N mineralization rates, or specific N loss pathways. Therefore, explanations related to microbial regulation, nutrient transformation, and N retention should be considered potential mechanisms rather than directly demonstrated processes. Future studies incorporating microbial analyses, isotopic approaches, and direct measurements of N transformation and loss pathways are needed to clarify the mechanisms underlying improved N use under BSFF-based systems. Third, BSFF characteristics may vary depending on raw materials, processing methods, and production conditions. Differences in nutrient concentration, decomposition characteristics, and stability may influence its effectiveness as a fertilizer amendment. Therefore, establishing standardized BSFF quality parameters will be important for ensuring consistent performance and supporting wider agricultural adoption.

## 5. Conclusions

In this study, we demonstrate that integrating BSFF with mineral N fertilizer, particularly AS, effectively improves maize productivity, N uptake, and NUE under calcareous soil conditions. Among all evaluated treatments, ASBSFF consistently achieved the highest grain yield, biomass production, and NUE indices, highlighting the advantage of combining rapidly available mineral N with organic nutrient sources. The improved performance of ASBSFF was associated with enhanced nutrient availability, including NH_4_^+^-N, NO_3_^−^-N, available P, and available K, as well as the complementary effects of BSFF nutrient supply, organic carbon addition, and ammonium sulfate-induced improvement of N availability under alkaline soil conditions. Based on the two-year field experiment, applying 50% N from ammonium sulfate and 50% N from BSFF (total N rate of 180 kg N ha^−1^) is recommended as an effective nutrient management strategy for maize production in similar calcareous soils. However, the adoption of BSFF-based fertilization will depend on local availability, production capacity, processing infrastructure, and quality standardization. With the expansion of black soldier fly production through organic waste recycling systems, BSFF has the potential to become an accessible and sustainable fertilizer resource. Further multi-location and long-term studies are needed to evaluate economic feasibility, environmental impacts, and optimized BSFF application rates.

## Figures and Tables

**Table 1:** Initial soil physicochemical properties.

Property	Value
Total N (g kg^−1^)	0.8
Available P (mg kg^−1^)	8.5
Available K (mg kg^−1^)	63.01
SOC (g kg^−1^)	10.3
EC (dS m^−1^)	3.6
Soil pH	8.55

**Table 2:** Chemical properties of organic fertilizers.

Nutrient Content	Vermicompost	Black Soldier Fly Frass
Total N (%)	0.78	3.09
Total P (%)	0.85	2.47
Total K (%)	1.05	3.53
Total C (%)	18.35	35.77
C/N ratio	23.53	11.58
Moisture content (%)	37.3	34.8

Notes: Total N (nitrogen), total P (phosphorus), total K (potassium), and total C (carbon) concentrations were expressed on a dry-weight basis. Total N, total P, total K, and total C represent total nitrogen, total phosphorus, total potassium, and total carbon, respectively.

**Table 3:** Nutrient inputs supplied by organic amendments applied on an equivalent nitrogen basis.

Treatment	Organic Amendment Contribution	Amendment Applied (t ha^−1^)	N Input (kg ha^−1^)	P Input (kg ha^−1^)	K Input (kg ha^−1^)
BSFF	Sole BSFF	5.83	180	143.9	205.6
VC	Sole VC	23.07	180	196.2	242.3
ASBSFF	50% N from BSFF	2.91	90	71.95	102.8
ASVC	50% N from VC	11.54	90	98.1	121.2

Notes: Organic amendment application rates were calculated based on their total nitrogen concentrations to provide equivalent nitrogen inputs. Nutrient inputs were calculated by multiplying amendment application rates by their respective nutrient concentrations.

## Data Availability

The datasets generated during the current study are available from the corresponding author upon request. The data are not publicly available due to the fact that the reported experiment is an integrated part of a larger ongoing project. The data will be made publicly available after the larger project is complete.

## Supplementary Materials

The following supporting information can be downloaded at: https://www.mdpi.com/article/10.3390/plants15182786/s1, Table S1. Loadings and contributions of measured variables to the first two principal components.

## Abbreviations

The following abbreviations are used in this manuscript:

AE	Agronomic efficiency
ARE	Apparent recovery efficiency
AS	Ammonium sulfate
ASBSFF	Combination of AS and BSFF
ASVC	Combination of AS and VC
BSFF	Black soldier fly frass
C	Carbon
CEC	Cation exchange capacity
CK	Unfertilized control
EC	Electrical conductivity
K	Potassium
MSLA	Mean single leaf area
LAI	Leaf area index
N	Nitrogen
NUE	Nitrogen use efficiency
P	Phosphorus
PCA	Principal component analysis
PE	Physiological efficiency
SOC	Soil organic carbon
SPAD	Leaf chlorophyll content
U	Urea
UVC	Combination of U and VC
VC	Vermicompost
