# Supplementary material for: Black Soldier Fly Frass Combined with Ammonium Sulfate Enhances Maize Productivity and Nitrogen Use Efficiency Under Calcareous Soil Conditions
Source: Plants (Basel). 2026 Sep 11;15(18):2786. doi: 10.3390/plants15182786 (PMC13610737; doi:10.3390/plants15182786)
Supplement: Supplementary file 1 [file plants-15-02786-s001.zip › plants-4484695-supplementary.pdf]

plants

Table S1. Loadings and contributions of measured variables to the first two principal components.

1. **Variable**	2. **PC1 loading**	3. **PC2 loading**	4. **Contribution to PC1 (%)**	5. **Contribution to PC2 (%)**
6. Grain yield	7. 0.38	8. -0.05	9. 16.2	10. 0.3
11. Total biomass	12. 0.37	13. -0.12	14. 15.4	15. 1.8
16. Total N uptake	17. 0.39	18. -0.08	19. 17.1	20. 0.8
21. Available P	22. 0.34	23. 0.28	24. 12.5	25. 8.9
26. Available K	27. 0.36	28. 0.20	29. 14.0	30. 4.7
31. NH_4^+–N	32. 0.32	33. 0.35	34. 11.8	35. 13.5
36. NO_3^-–N	37. 0.33	38. 0.31	39. 12.2	40. 10.6
41. EC	42. 0.18	43. 0.55	44. 3.8	45. 32.1
46. Soil pH	47. -0.09	48. -0.58	49. 1.0	50. 27.3
51. **Eigenvalue**	52. **5.76**	53. **1.35**	54. **64.0**	55. **15.0**
